# Low-temperature direct copper-to-copper bonding enabled by creep on (111) surfaces of nanotwinned Cu

**DOI:** 10.1038/srep09734

**Published:** 2015-05-12

**Authors:** Chien-Min Liu, Han-Wen Lin, Yi-Sa Huang, Yi-Cheng Chu, Chih Chen, Dian-Rong Lyu, Kuan-Neng Chen, King-Ning Tu

**Affiliations:** 1 Department of Materials Science and Engineering, National Chiao Tung University, Hsinchu, 30010 Taiwan, R.O.C; 2 Department of Electronics Engineering, National Chiao Tung University, Hsinchu, 30010 Taiwan, R.O.C; 3 Department of Materials Science and Engineering, University of California at Los Angeles, Los Angeles, CA, 90095, U.S.A

## Abstract

Direct Cu-to-Cu bonding was achieved at temperatures of 150–250 °C using a compressive stress of 100 psi (0.69 MPa) held for 10–60 min at 10^−3^ torr. The key controlling parameter for direct bonding is rapid surface diffusion on (111) surface of Cu. Instead of using (111) oriented single crystal of Cu, oriented (111) texture of extremely high degree, exceeding 90%, was fabricated using the oriented nano-twin Cu. The bonded interface between two (111) surfaces forms a twist-type grain boundary. If the grain boundary has a low angle, it has a hexagonal network of screw dislocations. Such network image was obtained by plan-view transmission electron microscopy. A simple kinetic model of surface creep is presented; and the calculated and measured time of bonding is in reasonable agreement.

Direct Cu-to-Cu bonding is of wide interest because it has the potential to replace solder joint in high-end packaging technology, including the packaging of mainframe computers. Under ultra-high vacuum (UHV) conditions, direct Cu-to-Cu bonding can be achieved at room temperature; however, the Cu surface must be cleaned prior to bonding by using a surface-activated approach and the process is rather time-consuming^1^. In ordinary vacuum conditions on the order of 10^−3^ to 10^−4^ torr, direct Cu-to-Cu bonding can be obtained by thermal compression at temperatures exceeding 300 °C for more than 30 min under a compression of approximately 100 psi^2^,^3^,^4^. Nevertheless, the temperature is much higher than the melting temperature of eutectic SnPb and Pb-free SnAgCu solders. The typical reflow temperature of these solders is below 250 °C. Thus, the key challenge in direct Cu-to-Cu bonding is how to lower the bonding temperature to 250 °C in ordinary vacuum.

The essential process in direct bonding is found to be surface diffusion, where atoms may jump across the bonding interface or along the interface. As seen in Table 1, the surface diffusion coefficient on (111) surface of Cu is 3 to 4 orders of magnitude faster than that on other surfaces, indicating that the bonding temperature can be lowered on (111) surfaces. However, it is unrealistic to expect the use of (111) oriented single crystals of Cu in large-scale applications. To overcome this challenge, the surface of a (111) oriented single crystal of Cu is replaced by a highly [111]-oriented Cu polycrystalline thin film^5^,^6^,^7^. Furthermore, when an oriented nano-twin of Cu (nt-Cu) is deposited with the (111) twin plane parallel to the free surface, low temperature of direct bonding down to 150 °C can be achieved. To achieve direct Cu-to-Cu bonding, the critical requirement is that one of the Cu pieces to be bonded must have a (111) surface. The bonding can be carried out at a fast pace if both Cu pieces are <111> oriented and nt-Cu.

To verify the bonded interface, the formation of a twist-type grain boundary with a hexagonal network of dislocations was observed in plan-view TEM. The pull test showed that the bonded interface is stronger than the interface between Cu and its substrate. A kinetic model of surface creep under compression is presented to calculate the time of interfacial bonding and it is in reasonable agreement with experimental measurements.

## Results

**Figure 1:**
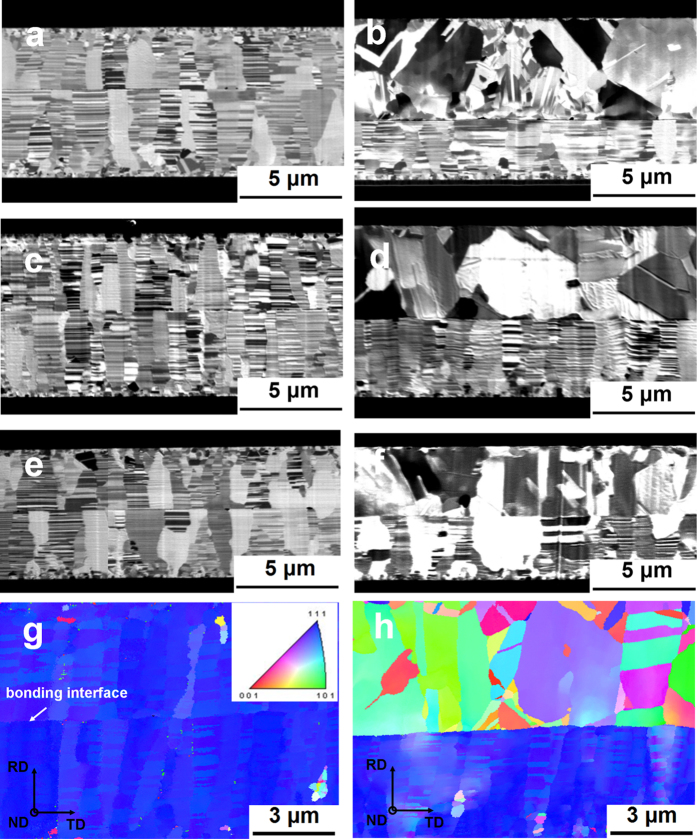
Bonding between two electroplated Cu films at various temperatures and durations. (**a**) FIB image of two (111) nt-Cu films bonded at 150 °C for 1 h. (**b**) FIB image of a randomly oriented Cu film bonded with a (111) nt-Cu film at 150 °C for 1 h. (**c**) FIB image of two (111) nt-Cu films bonded at 200 °C for 30 min. (**d**) FIB image of a randomly oriented Cu film bonded with a (111) nt-Cu at 200 °C for 30 min. (**e**) FIB image of two (111) nt-Cu films bonded at 250 °C for 10 min. (**f**) FIB image of a randomly oriented Cu film bonded with a (111) nt-Cu film at 250 °C for 10 min. (**g**) Cross-sectional EBSD orientation image of two (111) nt-Cu films bonded at 200 °C for 30 min. (**h**) Cross-sectional EBSD orientation image map of a randomly oriented Cu film bonded with a (111) nt-Cu at 200 °C for 30 min.

Figure 1a presents a cross-sectional FIB image of two electroplated nt-Cu films bonded at 150 °C for 1 h under a pressure of approximately 100 psi (0.69 MPa) in a vacuum of 10^−3^ torr. The thickness was measured as 3.0 and 4.3 μm for the top and bottom nt-Cu films, respectively. The small area of the bonded interface exhibits no voids, yet residual voids are present in the larger bonded interface, which will be demonstrated below. Direct bonding can also be achieved when a randomly oriented Cu film (top) is bonded to a (111)-oriented nt-Cu film (bottom). The bonded interface exhibited some small voids. The bonding time can be shortened to 30 min by increasing the bonding temperature to 200 °C. Fig. 1c presents the cross-sectional interface for nt-Cu bonded to nt-Cu at 200 °C for 30 min, which yields an interface that is almost free of residual voids. Bonding of (111)-oriented nt-Cu to ordinary Cu can also be achieved at this condition, as shown in Fig. 1d. The bonding time can be further reduced to less than 10 min by increasing the bonding temperature to 250 °C, as shown by the cross-sectional image in Fig. 1e. Almost no voids are present in the interface, and the nano-twinned columnar grains remain after the bonding process. The bonding of (111)-oriented nt-Cu to ordinary Cu at 250 °C yielded the results in Fig. 1f, in which very few voids were found.

EBSD was employed to verify the grain orientation of the films reported in Figs. 1c and 1d. Fig. 1g presents a rolling-direction (RD) orientation image map of the bonded interface of nt-Cu to nt-Cu at 200 °C for 30 min. The arrow in the figure indicates the bonding interface, and the inverse pole figure on the bottom left corner identifies the color coding display for the orientations in this study. The Cu grains near the bonding interface are shown in blue, indicating that all the grains were oriented in the <111> direction. Fig. 1h presents an EBSD orientation image map for the sample in Fig. 1d. The Cu grains for the ordinary Cu (top) appear to be randomly orientated, whereas most of the grains near the bonding interface are not <111> oriented. However, direct bonding was established between the (111)-oriented nt-Cu and the ordinary Cu piece.

The primary capability of the low-temperature bonding behavior is attributed to the presence of <111> -oriented Cu surfaces, which possess the highest surface diffusivity values among all the crystallographic planes of Cu^8^,^9^,^10^. Additionally, the oxidation rate of the Cu (111) surface is the slowest relative to that of the other planes^11^.

**Figure 2:**
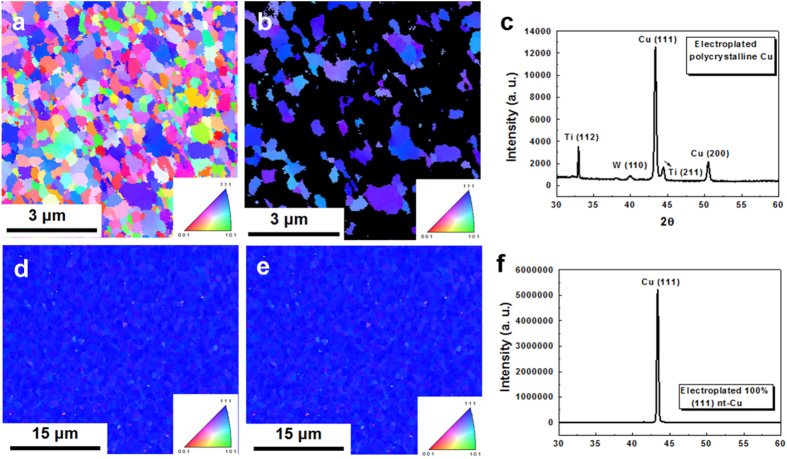
Orientation analysis of Cu films prepared by electroplating. (**a**) Plan-view EBSD orientation image map of the randomly oriented Cu film. (**b**) Plan-view EBSD orientation image map showing only the (111) Cu grains of Fig. 3(a). The grains with mis-orientation angles exceeding 15° relative to the [111] direction are shown in black. The (111) grains occupy 26% of the surface area. (**c**) XRD analysis of the randomly oriented Cu. (**d**) Plan-view EBSD orientation image map of a (111) nt-Cu film. (**e**) Plan-view EBSD orientation image map showing only the (111) Cu grains of Fig. 3(d). The grains with mis-orientation angles larger than 15° relative to the [111] direction are shown in black. The (111) grains occupy 100% of the surface area. (f) XRD analysis of the (111) nt-Cu film.

Figure 2a presents a plan-view EBSD orientation image map for ordinary electroplated Cu films. To calculate the percentage of <111> -oriented surfaces, the <111> -oriented Cu surface grains that reside within 15° relative to the normal of the (111) plane were defined as normal. Then, the remaining grains in Fig. 2a were marked in black. Fig. 2b presents only the (111)-oriented grains, which occupy 26% of the surface area. The corresponding X-ray diffraction pattern in Fig. 2c indicates that the intensity of the (111) reflection is 5.9 times larger than that of the (200) reflection. For standard powder diffraction, the intensity ratio of (111) peak to (200) peak is 2.2. As a result, the Cu film in Fig. 2a had a very weak (111) preferred orientation.

However, 100% of the surface grains had a (111) orientation in the electroplated nt-Cu film. Fig. 2d presents the EBSD orientation image map of nt-Cu films. Fig. 2e presents the <111> -oriented grains, illustrating that 100% of the surface was composed of <111> -oriented Cu grains. Fig. 2f presents the corresponding X-ray diffraction pattern for the nt-Cu film, which possessed an extremely high <111> -preferred orientation; accordingly, the Cu (200) reflection is invisible in this figure. The intensity of the (111) reflection is 7,412 times greater than that of the (200) reflection. The Cu atoms diffuse at a rate that is four orders of magnitude faster on (111) surfaces than on (200) surfaces at 150 °C (see Table 1). Therefore, direct bonding can be achieved with (111) surfaces at lower temperatures or at shorter times by employing the surface diffusion-controlled bonding process.

**Figure 3:**
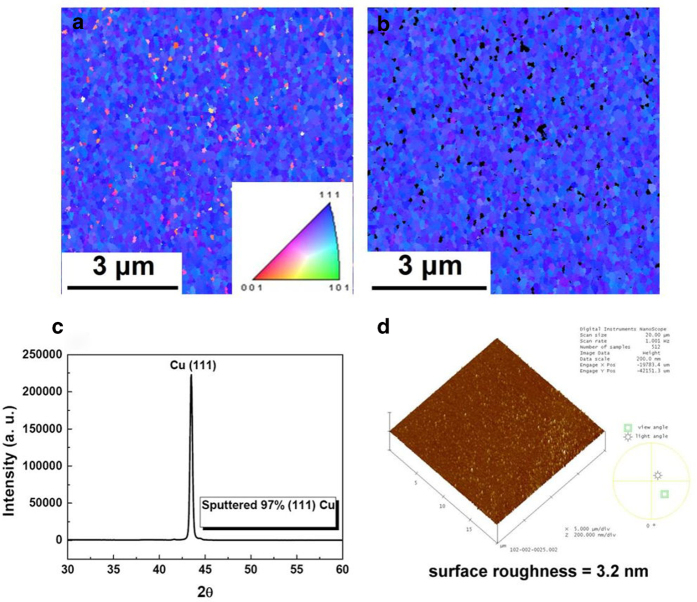
Characterization of the sputtered (111) Cu films. (**a**) Plan-view EBSD orientation image map of the (111) Cu surface. (**b**) Plan-view EBSD orientation image map showing only the (111) Cu grains in Fig. 4(a). The grains with mis-orientation angles exceeding 15° relative to the (111) direction are shown in black. The (111) grains occupy 97% of the surface area. (**c**) XRD analysis of the Cu film. (**d**) Surface roughness, measured by SPM, was 3.2 nm.

Figure 3 shows that the sputtered <111> -oriented Cu can also be employed to achieve low-temperature bonding. Fig. 3a shows the plan-view EBSD orientation map of the sputtered film. Fig. 3b shows only the (111) Cu grains in Fig. 3a, and 97% of the grains reside in the (111)-preferred orientation. Fig. 3c shows the corresponding X-ray diffraction pattern. Fig. 3d shows that the surface roughness is 3.2 nm.

**Figure 4:**
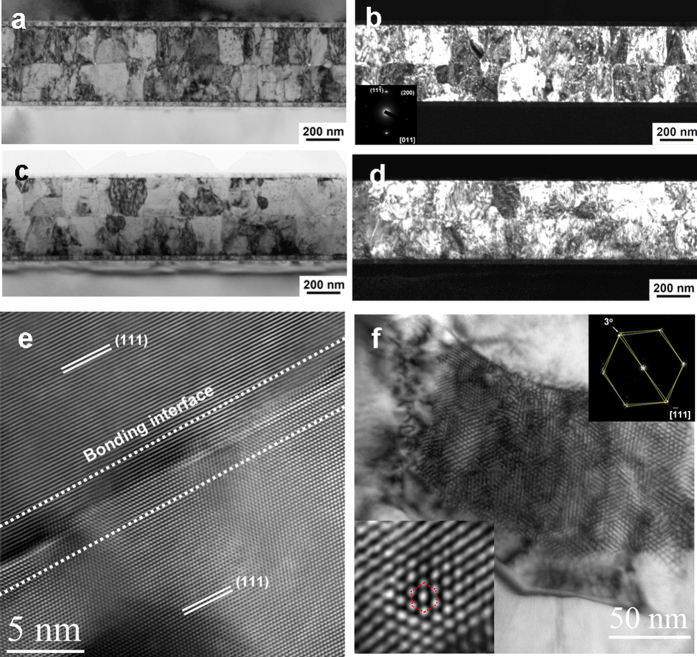
Bonding between two sputtered films with extremely high (111)-preferred orientations. The (111) grains occupy 97% of the bonding surface. (**a**) Bright-field TEM image of two (111) Cu thin films bonded at 150 °C for 1 h. (**b**) Dark-field TEM image obtained using the (111) diffraction for two (111) Cu thin films bonded at 150 °C for 1 h. (**c**) Bright-field TEM image of two (111) Cu thin films bonded at 200 °C for 30 min. (**d**) Dark-field TEM image of two (111) Cu thin films bonded at 200 °C for 30 min. (**e**) Cross-sectional HRTEM of the bonded interface after annealing 200 °C for 30 min. (**f**) Plan-view TEM image illustrating the hexagonal network of screw dislocations at the bonded interface. The left inset shows an enlarged image of the dislocation network, and the right inset presents the diffraction pattern obtained for the bicrystals. The rotational angle is 3°.

Figure 4a presents the bright-field TEM image of a bonded sample of two 0.2-μm-thick sputtered Cu films. Almost void-free bonding was achieved at 150 °C for 1 h. The dark-field TEM image in Fig. 4b indicates that the majority of the Cu grains were <111> oriented and that the grains were columnar. The corresponding diffraction pattern is shown in the bottom-left corner of Fig. 4b. The necessary bonding time decreased to 30 min as the bonding temperature was increased to 200 °C, as illustrated in Figs. 4c and 4d. No significant grain growth was observed in any of the above conditions.

**Figure 5:**
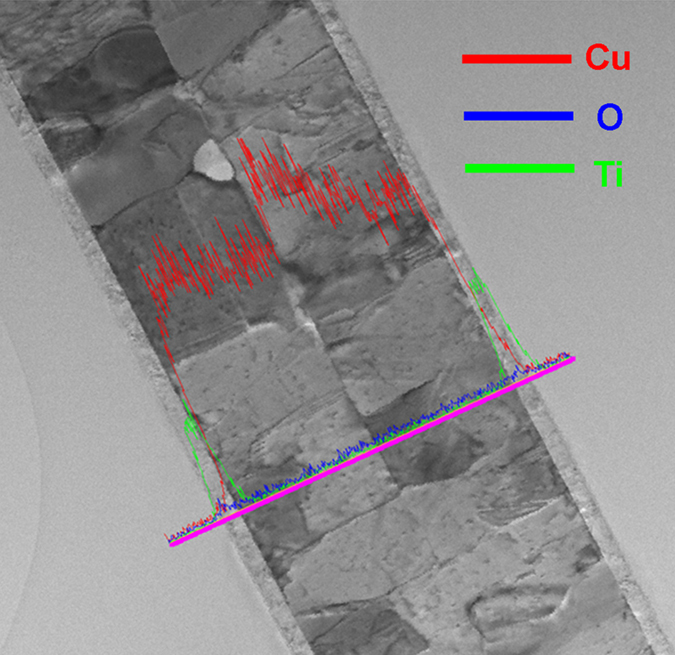
TEM EDS line-scan across the entire sample of two 97% (111) Cu thin films, bonded at 200 °C for 30 min.

Figure 4e presents a high-resolution cross-sectional TEM image of the bond interface, in which the lattice of Cu is clearly visible. No amorphous layer was observed at this interface. Together, these findings indicate that the interface was continuous on the atomic scale. A twist-type grain boundary can be seen when two <111> planes join to form a grain boundary. If the twist angle is small, a hexagonal dislocation network can be observed in a corresponding plan-view bright-field TEM image^12^, as shown in Fig. 4f, which was obtained from a sample bonded at 200 °C for 30 min. The lower-left inset presents a magnified image of the hexagonal network. The rotational angle is 3° , as revealed by the diffraction pattern in the upper-right inset. Six small satellite spots surround each 220 spot due to double diffraction of the bicrystals. Fig. 5 shows a line-scan across the bonded interface recorded by EDS. As can be seen, the interface is almost oxide-free.

**Figure 6:**
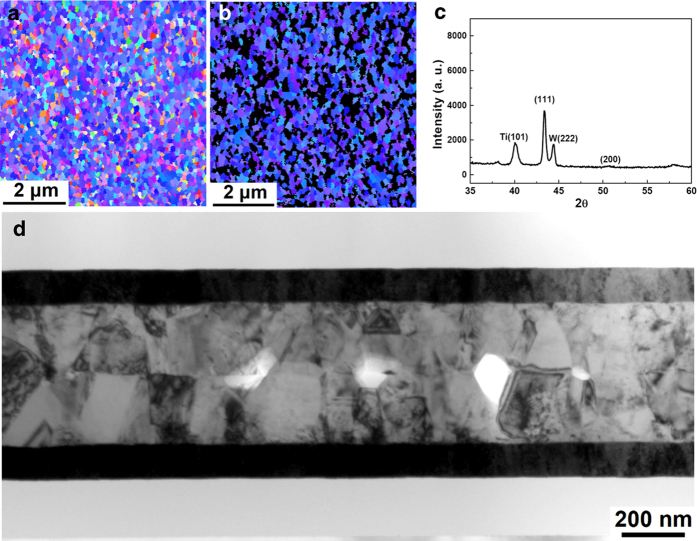
Characterization of sputtered 64% (111) Cu films on a TiW layer and bonding interfaces. (**a**) Plan-view EBSD orientation image map of the 64% (111) Cu surface. (**b**) Plan-view EBSD orientation image map showing only the (111) Cu grains in Fig. 7(a). The grains with mis-orientation angles exceeding 15° relative to the (111) direction are shown in black. The (111) grains occupy 64% of the surface area. (**c**) XRD analysis of the Cu film. (**d**) Cross-sectional TEM image showing the bonding interface after 200 °C for 30 min.

Figure 6 shows the bonding between two sputtered Cu films in which 64% of the surfaces were (111) oriented. Fig. 6a shows the EBSD orientation map of the film, and Fig. 6b shows the EBSD map of only the (111) Cu grain in Fig. 6a. Fig. 6c shows the corresponding X-ray diffraction pattern. Fig. 6d shows the cross-sectional TEM image of the bonded interface with voids. When two electroplated Cu films with surfaces having 26% of grains in the (111) orientation were bonded, they would eventually de-bond, even after bonding at 200 °C for 1 h. The above results suggest that the rapid diffusivity of the Cu (111) surface plays a critical role in the low-temperature direct bonding of Cu.

**Figure 7:**
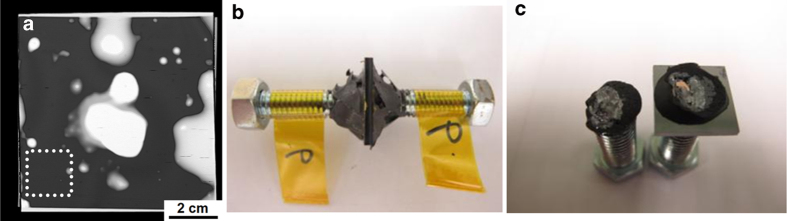
Bonding of 8 × 8-cm^2^ samples of sputtered 97% (111) Cu films and the corresponding tensile test results. (**a**) Scanning acoustic microscopy image obtained after 200 °C bonding for 1 h. The dark region covering approximately 82% of the area indicates a good bonding interface without voids, whereas the white region represents the bonding interface with voids. The white dotted 2 × 2-cm^2^ square in the figure represents the location from which one specimen was cut and removed. (**b**) The specimen from the dotted square in (**a**) after the tensile test. No fracture occurred in this specimen when loaded to the limits of the tensile machine (175 kg). (**c**) Photograph showing the fracture morphology after the tensile test for a different specimen. Most of the fracturing occurred at the Si interphase instead of the Cu-Cu bonding interface.

Additionally, tensile tests were performed on 2 × 2 cm bonded specimens, demonstrating that the bonded interface possesses high strength. Three of the 2 × 2 cm specimens were found to survive tensile testing in excess of 175 kg, which is equivalent to 4.3 MPa. The other two specimens fractured at 2.7 MPa and 1.3 MPa. These two specimens may have less bonding area than the three non-failed ones. The scanning acoustic microscopic (SAT) results are presented in Fig. 7a, in which the dark region indicates the achievement of a good bonding interface without voids, whereas the white region represents a bonding interface with voids. Fig. 7b shows the sample taken from the dotted area in Fig. 7a, and no fracture occurred in this sample under a tensile load of 175 kg. Fig. 7c shows a fractured sample, in which the fracture occurred at the Si-Cu interface rather than the Cu-Cu bonding interface, indicating that the Cu-Cu bonding interface is quite strong.

Surface roughness also plays a critical role in direct Cu bonding. The typical surface roughness for Cu films ranges from 1.0 to a few nanometers. The direct bonding of Cu can be achieved at lower temperatures for smaller surface roughness values^13^,^14^. The surface roughness values were 3.2, 6.5, and 11.6 nm for the sputtered (111) Cu, electroplated (111) Cu, and randomly oriented Cu films, respectively, in this study. Therefore, the bonding temperature and time can be further reduced if the standard chemical-mechanical polish is adopted to flatten the surface of the electroplated Cu films.

## Discussion

**Figure 8:**
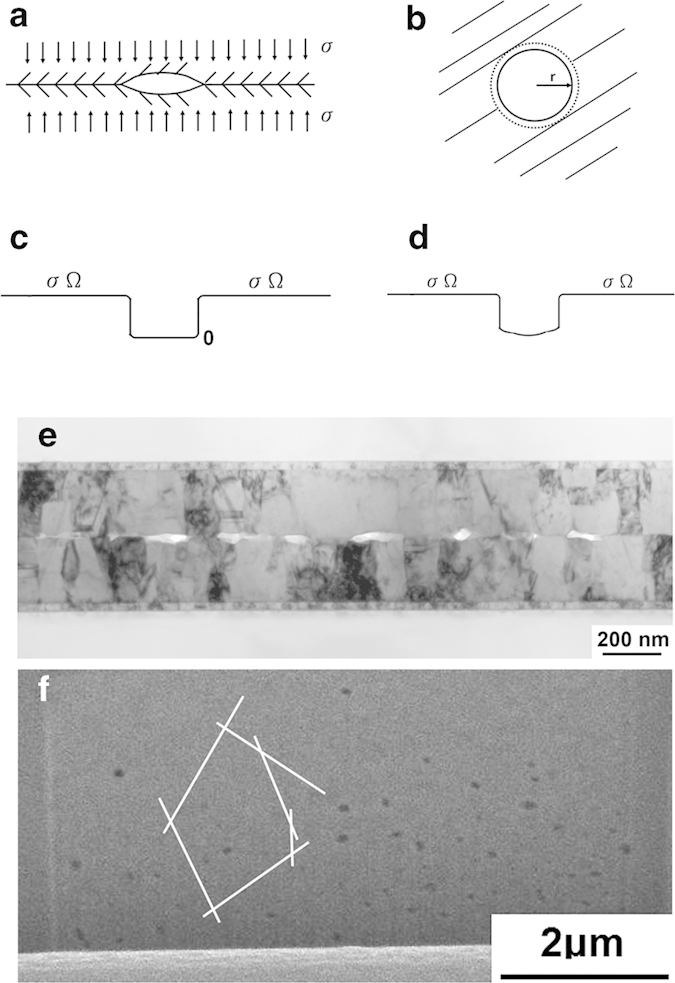
Schematic drawing of bonding mechanism. (**a**) Cross-sectional view of a cavity under thermal compression. (**b**) Plan view of a cavity under thermal compression. (**c**) Stress potential across the cavity. (**d**) Consideration of the Laplace pressure on the cavity. (**e**) Cross-sectional TEM image presenting the remaining cavities within the interface after bonding at 200 °C for 10 min. (**f**) FIB ion image showing the plan view of residual void distribution in the sample annealed at 200 °C for 30 min.

To analyze the kinetics of direct bonding, the cross-sectional view and top view of a small region of the Cu-Cu interface containing a circular cavity under thermal compression are presented in Figs. 8a,b, respectively. A distribution of cavities in the interface is assumed. A cavity of radius *r* is considered here. The matrix surrounding the cavity is assumed to be under a compressive stress of *σ*. The stress applied in this study was in the elastic regime. The free surface of the cavity has no normal stress. Thus, the stress potential drops between the edge and center of the cavity, as depicted in Fig. 8c. This stress potential drop must be a gradual phenomenon. Except for the drop, no potential gradient exists relative to the outside and inside of the cavity under the assumption that there is no stress center in the compressive region. According to Fick’s first law, when there is no potential or concentration gradient, no diffusion or interdiffusion flux occurs except random walk.

The cavity has two curvatures: one is indicated by the radius *r* in Fig. 8b, and the other is derived from the two surfaces of the cavity, which are of radius *R*. The Laplace pressure of both curvatures tends to exert tensile stress onto the matrix atoms surrounding the cavity. Added to the analysis is the potential due to the Laplace pressure, 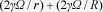, as shown in Fig. 8d. When the free surface has no normal stress, the surface atoms on a curved surface experience the same Laplace pressure potential as the atoms within the matrix.

To analyze the kinetics, a diffusion field was set up in cylindrical coordinates, and the field was then solved with known boundary conditions. This approach can yield a complicated process because the cavity has a curved surface. Diffusion on a cusp surface obeys a fourth-order diffusion equation. However, the problem is simplified by using the Nabarro-Herring (or Coble) model of creep, as shown below^15^. If either the upper or lower surface of the cavity is assumed to be a (111) surface, then diffusion occurs mainly on this surface because the <111> orientation remains after one surface atomic layer is added to the (111) surface. This process will then proceed to close the cavity for direct bonding. The surface atomic flux is given as
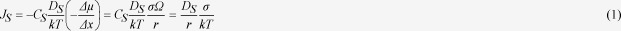
where 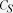 is the surface atomic concentration, 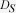 is the surface diffusivity, *σ* is the applied compressive stress, *kT* is the thermal energy, Ω is the atomic or vacancy volume, and *r* is the radius of the cavity, as shown in Fig. 8b, which is taken as the distance of surface diffusion. If the unit of surface atomic flux is assumed to be 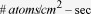 rather than 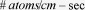, then 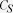 can be taken as the number of atoms in an atomic layer on the surface, which yields 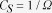 for a pure element.

To calculate the stress potential *σΩ*, pressure is applied on the order of 100 psi during direct bonding, and the atomic volume of the Cu atom was taken as 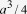, where 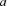 = 0.3615 nm is the Cu lattice parameter. When 146 psi 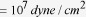, 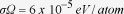 can be obtained. At 200 °C, 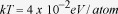. Thus, 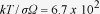.

The time needed to achieve direct bonding can be estimated as the time needed to fill the cavity, which has a volume of *V*. To simplify volume calculation, the shape of the cavity is replaced by a pancake-shaped void, where the radius is that of the cavity with a thickness of m atomic layers of (111) Cu. Thus,
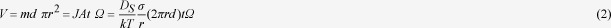
where *d* is the atomic height of a Cu atom or the interplanar spacing of the Cu (111) plane, which gives *d = *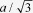. Additionally, m is the number of (111) planes in the cavity, *r* is the radius of the cavity (see Fig. 8b), *A = 2πrd* is the cross-sectional area for the surface diffusion of atoms entering the cavity, and *t* is the time to remove all the vacancies in the pancake-shaped cavity.
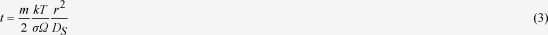
The surface diffusivity, 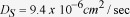, and 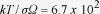 are taken from Table 1 for estimating the time required for bond formation at 200 °C. For the size of the cavity, Fig. 8e presents a cross-sectional TEM image of the cavities that developed after bonding at 200 °C for 10 min. The average size of the cavities is 86 nm in width and 22 nm in height. Take *r* = 43 nm; then for *m*, *md* = 22 nm. Again take *m* = 110, and *t *≈ 0.1 sec can be obtained from Eq. (3). As shown in Figs. 1 and 4, the bonding time measured during the experiments at 200 °C was 30 min, but after 10 min was taken away, the time became 20 min, or 1,200 sec. Thus, the discrepancy obtained is approximately four orders of magnitude. Although the calculated and experimental results do not appear to correspond well, such discrepancy is expected because the calculations involved an extremely fast diffusion over a very short distance. The weakness of the above model is that it does not consider the source of the atoms needed to fill the cavity. The atoms must diffuse from the bonded region, which is a short distance away from the edge of the cavity, as indicated by the broken circle in Fig. 8b. Assuming that the diffusion between the broken and solid circles is governed by grain boundary diffusion, the diffusion rate is several orders of magnitude slower than the theoretical diffusivity on a (111) surface. A more detailed analysis would consider both types of diffusion by incorporating a simulation, but such an effort is beyond the scope of this paper.

To minimize the extent of the above discrepancy, FIB is employed to obtain the residual void distribution in the sample annealed at 200 °C for 30 min, as shown in Fig. 8f. The FIB cuts a surface with a small tapered angle relative to the bonded interface, i.e., the normal of the cut surface is offset relative to the normal of the bonded interface by a very small angle. A distribution of residual voids can be observed in the bonded interface, as shown in Fig. 8f. According to the Wigner-Seitz approximation^16^, by bisecting the centers of two neighboring residual voids, cells are formed, one of which is indicated by the white lines in Fig. 8f. A circle is placed in a cell, and this circle is then assumed to be the size of the cavity in the initial stage of bonding. To calculate the bonding time, a big circle of *r *≈ 1 μm should be chosen. To establish the appropriate thickness of the cavity, the central part should initially be thicker than the edge region. For comparison, take *md* = 22 nm, as considered above. The calculated time is then 40 sec, which presents a better agreement with the measured time of 1,800 sec. The experimentally measured surface diffusivity on Cu surfaces at 200 °C is approximately
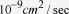
17, which is four orders of magnitude smaller than the theoretical surface diffusivity used in the above calculation. Hence, the discrepancy is primarily due to the unrealistic surface diffusivity (in UHV rather than in ordinary vacuum) used in the calculation. If an intermediate value of 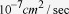 is taken, then the calculated and experimental results correspond very well.

The above analyses reveal an important insight regarding the direct bonding process: the rate of cavity shrinkage decreases with decreasing size. The diffusivity on the nanosphere surface is significantly reduced as the radius of the cavity decreases. This trend is apparent in Table 1, which illustrates the significant variation of the surface diffusivity with respect to orientation. Furthermore, grain boundary diffusion dominates, which is why many residual voids survive within the bonded interface^18^. However, the small residual voids do not have a significant effect on the bonding strength.

The power law of creep has been employed to analyze direct bonding^19^,^20^,^21^. Whether this approach can improve the above analysis for low-temperature direct bonding is unclear. However, the power law of creep tends to be empirical. The exponential factor “*n*” of stress in the power law 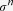 is ill-defined. This topic should be investigated further in future research.

Equation (3) illustrates that the bonding time is inversely proportional to the applied stress and surface diffusivity. With decrease in surface diffusivity by three orders of magnitude relative to that on the (111) surface when diffusing on the (100) or (110) surface, as shown in Table 1, the bonding time must increase by three orders of magnitude when all other parameters are held constant. Therefore, bonding would require 30,000 min at 200 °C on either the (100) or (110) surface, which is clearly impractical. This result demonstrates that the critical condition for direct bonding is fast surface diffusion. The effect of temperature on bonding is mainly due to surface diffusivity. The diffusivity on the (111) surface decreases by a factor of 14 when reducing the temperature from 250 °C to 150 °C, and then, according to Table 1, the time allowed for bonding must be increased from 10 min to 1 h, which is an increase by a factor of six. Nevertheless, direct bonding can occur at 150 °C because the diffusion profile has a Gaussian distribution. Regarding Cu surface diffusion, electromigration within Cu interconnects occurs by surface diffusion at the device operating temperature of 100 °C. Furthermore, electromigration has been shown to occur faster on the (111) surface of Cu than on other surfaces.

Cu oxides may form on the surfaces of the Cu films before bonding. The Cu films were exposed to the air at room temperature for approximate 5 minutes before they were put in the vacuum. Thus, a very thin layer of Cu oxides may form on the surface of the Cu film. However, during the bonding process, the Cu films were under compression, the surface roughness will break the surface oxide into pieces and allow pure Cu atoms to meet and bond as well as to enhance the dissolution of oxygen into Cu, because there is a certain solubility of oxygen in Cu. As shown in Fig. 5, oxygen signals were not detected in the bonded interface. Therefore, we speculate that the Cu oxides might decompose at the bonding temperatures under the vacuum level of 10^−3^ torr. Then the oxygen atoms may dissolve into the Cu films. Yet, further study needs to be done to investigate the potential impact of the surface oxides on the Cu direct bonding.

## Conclusions

In summary, by using the <111> -oriented nt-Cu obtained either by electroplating or by sputtering, direct Cu-to-Cu bonding can be experimentally achieved at low temperatures, ranging from 150 °C to 250 °C, in an ordinary vacuum of 10^−3^ torr, and under a pressure of 100 psi for 10–60 min. This achievement is verified by conducting systematic experimental bonding studies as a function of time and temperature. Plan-view and cross-sectional imaging of the bonded interfaces were performed. In cross-sectional TEM, certain bonded interfaces form twist-type low-angle grain boundaries with a hexagonal network of dislocations. The most critical parameter for direct bonding is fast surface diffusivity on the (111) surface of Cu. A simple creep model is proposed to account for this observation. The agreement between the calculated and measured times of direct bonding is reasonable.

## Methods

### Preparation of Cu films

In this study, four types of Cu films for Cu-Cu direct bonding were prepared. They were sputtered 97% (111)-oriented Cu films, sputtered 64% (111)-oriented Cu films, electroplated (111)-oriented nano-twinned-Cu (nt-Cu) films, and electroplated randomly oriented Cu films.

To fabricate the sputtered 97% (111)-oriented Cu film, a 100 nm-thick Ti adhesion layer and a 200 nm-thick Cu film were sequentially sputtered on an 8-in Si wafer using an Oerlikon Cluster Line 300. The Cu film has a very strong (111)-preferred orientation^22^. The results from electron backscattered diffraction (EBSD) indicate that 97% of the film surface was in the (111) orientation. The average surface roughness of the sputtered film was 3.2 nm.

When the Ti adhesion layer was replaced by TiW at the same thickness, the sputtered Cu films exhibited fewer (111) surface grains. The (111) grains occupied 64% of the surface area.

To fabricate the electroplated (111)-oriented nt-Cu film, a high-purity CuSO_4_ solution was used as the electrolyte and a high-purity (99.99%) copper sheet was employed as the cathode. Appropriate surfactants and 40 ppm HCl were added to the electrolyte. The use of an appropriate electrolyte stirring speed is essential during electroplating. A speed of 900 rpm was achieved using a magnetic stirring rod. The purpose of stirring is the creation of a strong Cu ion shear flow relative to the deposition surface, which triggers the formation of nt-Cu. To fabricate the substrate, 100 nm of Ti was sputtered onto a Si wafer as the adhesion layer, followed by the sputtering of a 200-nm-thick Cu seed layer. The Si substrate was cut into 3 × 1 cm^2^ pieces, which were immersed in the electrolyte during electroplating. The applied DC current density was 80 mA/cm^2^. The deposition rate was 16 nm/s at this condition for a stirring rate of 900 rpm. After electrodeposition, the Cu films were electropolished at 1.75 V for 10 min in a solution of acetic acid (2.8 ml), glycerol (3 ml), and phosphoric acid (300 ml). All the surface Cu grains were (111) oriented. The average surface roughness of the nt-Cu film was 6.5 nm.

The electroplated randomly oriented Cu films were fabricated by DC electroplating. The electrolyte was prepared in the same manner except that no surfactants were added to the solution. The Cu films were also electropolished using the same conditions and solution described above. The average surface roughness of the randomly oriented film was 11.6 nm. The above samples were stored in a dry box before cleaning processes. Some samples were stored there over three months between fabrication and bonding.

### Direct bonding of Cu films

To bond the sputtered films, the commercial bonding tool EVG520HE (Electronic Visions EVG, Australia) was employed. Because this machine can bond samples larger than 8 × 8 cm^2^, the 8 inch wafers were cut into large pieces measuring 8 × 8 cm^2^. The bonding pressure was set to 100 psi in a vacuum of 1 × 10^−3^ torr. The bonding conditions were 250 °C for 10 min, 200 °C for 30 min, 200 °C for 1 h, and 150 °C for 1 h.

To bond the electroplated Cu films, the samples were cut into 3 × 3 mm^2^ pieces. Then, the pieces were cleaned ultrasonically in acetone for 5 min, dried with a N_2_ purge, cleaned with a mixed solution of HCl and deionized water (DI) water at the ratio of 1:1 for 30 sec, rinsed with DI water, and purged with N_2_ gas again. Next, the Cu films were placed face to face and fastened using a screw clamp. The bonding pressure at room temperature was measured to be 114 psi using a mechanical gauge. This alignment process took about 5 min. The specimens were loaded into a quartz furnace tube, to which vacuum was applied using a mechanical pump. The pressure was approximately 1 × 10^−3^ torr. The furnace was heated to the desired temperature over the necessary time period for a ramping rate of 1.3 °C/sec, and all cooling events were carried out by simply allowing the furnace to cool naturally. The reason for using different a bonding method for the electroplated Cu films is as follows. To electroplate (111)-oriented Cu films, we need to apply a high current density of 80 mA/cm^2^ on the sample. Due to the limitation of power in the current source used, we can electroplate samples with an area up to 3 × 1 cm^2^. Thus, the electroplated Cu samples cannot be bonded by the EVG520HE bonding tool, which requires a minimum area of 8 × 8 cm^2^.

### Examination of bonded interfaces

After bonding, the quality of the bonded interface was examined using a Hitachi scanning acoustic microscope (SAT, Model FS300II; Hitachi, Japan). The SAT examinations were performed in excess of 20 kHz with a point-to-point resolution of 10 nm. The principle of detection in SAT is ultrasonic transmission to the sample, followed by analysis of the reflected and transmitted ultrasonic waves with software. During SAT examination, the sample was placed in water because the ultrasonic waves are very sensitive to air.

For tensile tests, the wafers were cut into 2 × 2 cm^2^ squares. The tensile tests were performed using an auto inserting Pulling Force Tester 1220 S (SE Testsystems Co., Taiwan). Before tensile testing, each sample was adhered to a screw by applying a thermosetting resin adhesive at 150 °C for 2 h. After curing, the sample was placed in the auto-inserting pulling force tester. The strain rate is 10 mm/min. Only the sputtered films were tensile tested because of the limitation of the sample size for the electroplated films as described above. It is noteworthy that the curing process should not change the microstructures of the bonded specimens. Because according to our results, no grain growth was observed after annealing at 250 °C for 1 h. However, grain growth took place after 300 °C for 1 h. This may be attributed to the high thermal stability of the columnar (111) grains.

The microstructures of the bonded Cu-Cu interfaces were examined with a JEOL-2100 scanning transmission electron microscope (STEM). The TEM examinations were performed at 200 kV with a point-to-point resolution of 0.23 nm and a lattice resolution of 0.14 nm. Energy-dispersive X-ray spectrometer (EDS) analysis was performed on a Link ISIS 300 EDS attached to the JEOL-2100 TEM for determining the compositional distribution of oxidation within the local areas of the samples in the Cu-bonded layers. A focused ion beam (FIB) was employed to observe the bonding interface. X-ray diffraction and electron backscattered diffraction (EBSD) were utilized to analyze the orientations and grain orientations of the sputtered Cu films, nt-Cu films, and randomly oriented Cu films, respectively. The EBSD measurements were performed using a JEOL 7001 F field-emission scanning electron microscope (SEM) with an EDAX/TSL system operated at 25 kV. OIM^TM^ software was employed to analyze the orientation maps and crystallographic textures according to Kikuchi patterns. The surface roughness values of Cu films were measured using scanning probe microscopy (SPM, Veeco Dimension 3100).

## Additional Information

**How to cite this article**: Liu, C.-M. *et al*. Low-temperature direct copper-to-copper bonding enabled by creep on (111) surfaces of nanotwinned Cu. *Sci. Rep.* **5**, 9734; doi: 10.1038/srep09734 (2015).

## Figures and Tables

**Table 1:** Calculated Cu surface diffusivity on (111), (100), and (110) planes at various temperatures, ranging from 150 °C to 300 °C

**D**_**Surf.**_**\Temp.**	**(111)**	**(100)**	**(110)**
300 ^o^C	1.51 × 10^−5^	1.48 × 10^−8^	1.55 × 10^−9^
250 ^o^C	1.22 × 10^−5^	4.74 × 10^−9^	3.56 × 10^−10^
200 ^o^C	9.42 × 10^−6^	1.19 × 10^−9^	5.98 × 10^−11^
150 ^o^C	6.85 × 10^−6^	2.15 × 10^−10^	6.61 × 10^−12^

Surface Diffusivity (cm^2^/sec).

The parameters were taken from Reference 8
